# Development of a Single-Cycle Infectious SARS-CoV-2 Virus Replicon Particle System for Use in Biosafety Level 2 Laboratories

**DOI:** 10.1128/jvi.01837-21

**Published:** 2022-02-09

**Authors:** Johnny Malicoat, Senthamizharasi Manivasagam, Sonia Zuñiga, Isabel Sola, Dianne McCabe, Lijun Rong, Stanley Perlman, Luis Enjuanes, Balaji Manicassamy

**Affiliations:** a Department of Microbiology and Immunology, University of Iowa, Iowa City, Iowa, USA; b Coronavirus Laboratory, Departamento Biologia Molecular y Celular, Centro Nacional de Biotecnologia (CNB-CSIC), Madrid, Spain; c Department of Microbiology and Immunology, University of Illinois at Chicago, Chicago, Illinois, USA; University of North Carolina at Chapel Hill

**Keywords:** replicon, SARS-CoV-2

## Abstract

Research activities with infectious severe acute respiratory syndrome coronavirus 2 (SARS-CoV-2) are currently permitted only under biosafety level 3 (BSL3) containment. Here, we report the development of a single-cycle infectious SARS-CoV-2 virus replicon particle (VRP) system with a luciferase and green fluorescent protein (GFP) dual reporter that can be safely handled in BSL2 laboratories to study SARS-CoV-2 biology. The spike (S) gene of SARS-CoV-2 encodes the envelope glycoprotein, which is essential for mediating infection of new host cells. Through deletion and replacement of this essential S gene with a luciferase and GFP dual reporter, we have generated a conditional SARS-CoV-2 mutant (ΔS-VRP) that produces infectious particles only in cells expressing a viral envelope glycoprotein of choice. Interestingly, we observed more efficient production of infectious particles in cells expressing vesicular stomatitis virus (VSV) glycoprotein G [ΔS-VRP(G)] than in cells expressing other viral glycoproteins, including S. We confirmed that infection from ΔS-VRP(G) is limited to a single round and can be neutralized by anti-VSV serum. In our studies with ΔS-VRP(G), we observed robust expression of both luciferase and GFP reporters in various human and murine cell types, demonstrating that a broad variety of cells can support intracellular replication of SARS-CoV-2. In addition, treatment of ΔS-VRP(G)-infected cells with either of the anti-CoV drugs remdesivir (nucleoside analog) and GC376 (CoV 3CL protease inhibitor) resulted in a robust decrease in both luciferase and GFP expression in a drug dose- and cell-type-dependent manner. Taken together, our findings show that we have developed a single-cycle infectious SARS-CoV-2 VRP system that serves as a versatile platform to study SARS-CoV-2 intracellular biology and to perform high-throughput screening of antiviral drugs under BSL2 containment.

**IMPORTANCE** Due to the highly contagious nature of SARS-CoV-2 and the lack of immunity in the human population, research on SARS-CoV-2 has been restricted to biosafety level 3 laboratories. This has greatly limited participation of the broader scientific community in SARS-CoV-2 research and thus has hindered the development of vaccines and antiviral drugs. By deleting the essential spike gene in the viral genome, we have developed a conditional mutant of SARS-CoV-2 with luciferase and fluorescent reporters, which can be safely used under biosafety level 2 conditions. Our single-cycle infectious SARS-CoV-2 virus replicon system can serve as a versatile platform to study SARS-CoV-2 intracellular biology and to perform high-throughput screening of antiviral drugs under BSL2 containment.

## INTRODUCTION

Due to the highly contagious nature of severe acute respiratory syndrome coronavirus 2 (SARS-CoV-2) and the lack of sufficient immunity in the population, research on SARS-CoV-2 is permitted only under biosafety level 3 (BSL3) containment, which significantly limits SARS-CoV-2 research, i.e., only to institutions with BSL3 infrastructure. In addition, due to physical limitations, high-throughput screening of antiviral drugs can be impractical under BSL3 containment (1, 2). Fortunately, research with attenuated or conditional mutants of BSL3/BSL4 pathogens is allowed under lower containment upon demonstration of attenuation and safety (3). These include low-pathogenicity H5N1 (lacking the multibasic site in hemagglutinin [HA]), an Ebola virus conditional mutant (VP30 deletion mutant), and Yersinia pestis conditional mutants (4–6). Thus, to enable studies with SARS-CoV-2 under BSL2 containment, SARS-CoV-2 spike (S)-pseudotyped HIV lentivirus particles or recombinant vesicular stomatitis virus (VSV) with the native G gene replaced with the S gene have been developed and can be used to study the viral entry process and to identify viral entry inhibitors (7–9). In addition, several SARS-CoV-2 replicon reporter systems that retain the minimal viral genes necessary for intracellular replication have been developed (10–12). Moreover, conditional deletion mutants of SARS-CoV-2 capable of replicating only in complemented cells expressing viral proteins have been reported (N gene or open reading frame 3a or E [ORF3a/E] gene deleted) (13, 14). A recent study reported a single-cycle infectious particle system through coexpression of viral S, M, E, and N proteins along with a luciferase reporter carrying *cis*-acting elements (packaging sequences) of SARS-CoV-2 in the 3′ untranslated region (UTR) (15). The generation of these SARS-CoV-2 tools has allowed us to safely study various aspects of SARS-CoV-2 biology under BSL2 containment.

Here, we report the development of a single-cycle infectious SARS-CoV-2 virus replicon particle system (ΔS-VRP) with a luciferase and green fluorescent protein (GFP) reporter. Using a bacterial artificial chromosome-based reverse genetics system, we replaced the essential spike gene of SARS-CoV-2 with a luciferase (Luc) and GFP dual reporter (ΔS-Luc-GFP) (16). Cotransfection of the ΔS-Luc-GFP bacmid with a VSV-G-expressing plasmid resulted in efficient production of infectious VRPs [ΔS-VRP(G)], which can be further amplified in VSV-G-transfected cells. As expected, control vector-transfected cells failed to produce any infectious ΔS-VRPs, demonstrating that VRP infection is restricted to a single cycle. ΔS-VRP(G) stocks produced from VSV-G-expressing cells showed robust infection and expression of both reporters in various murine and human cell lines, indicating that various cell types are permissive to SARS-CoV-2 replication. Importantly, treatment of ΔS-VRP(G)-infected cells with either of the antivirals remdesivir (nucleoside analog) and GC376 (CoV 3CL protease inhibitor) resulted in a robust and drug dose-dependent decrease in both luciferase and GFP reporter activity, demonstrating that this platform can be useful for antiviral drug screening. Taken together, our studies demonstrate that this ΔS-VRP system with a dual reporter can serve as a versatile tool to investigate SARS-CoV-2 biology and perform antiviral drug screening under BSL2 containment.

## RESULTS

### Design of a single-cycle infectious SARS-CoV-2 virus replicon particle system.

**FIG 1:**
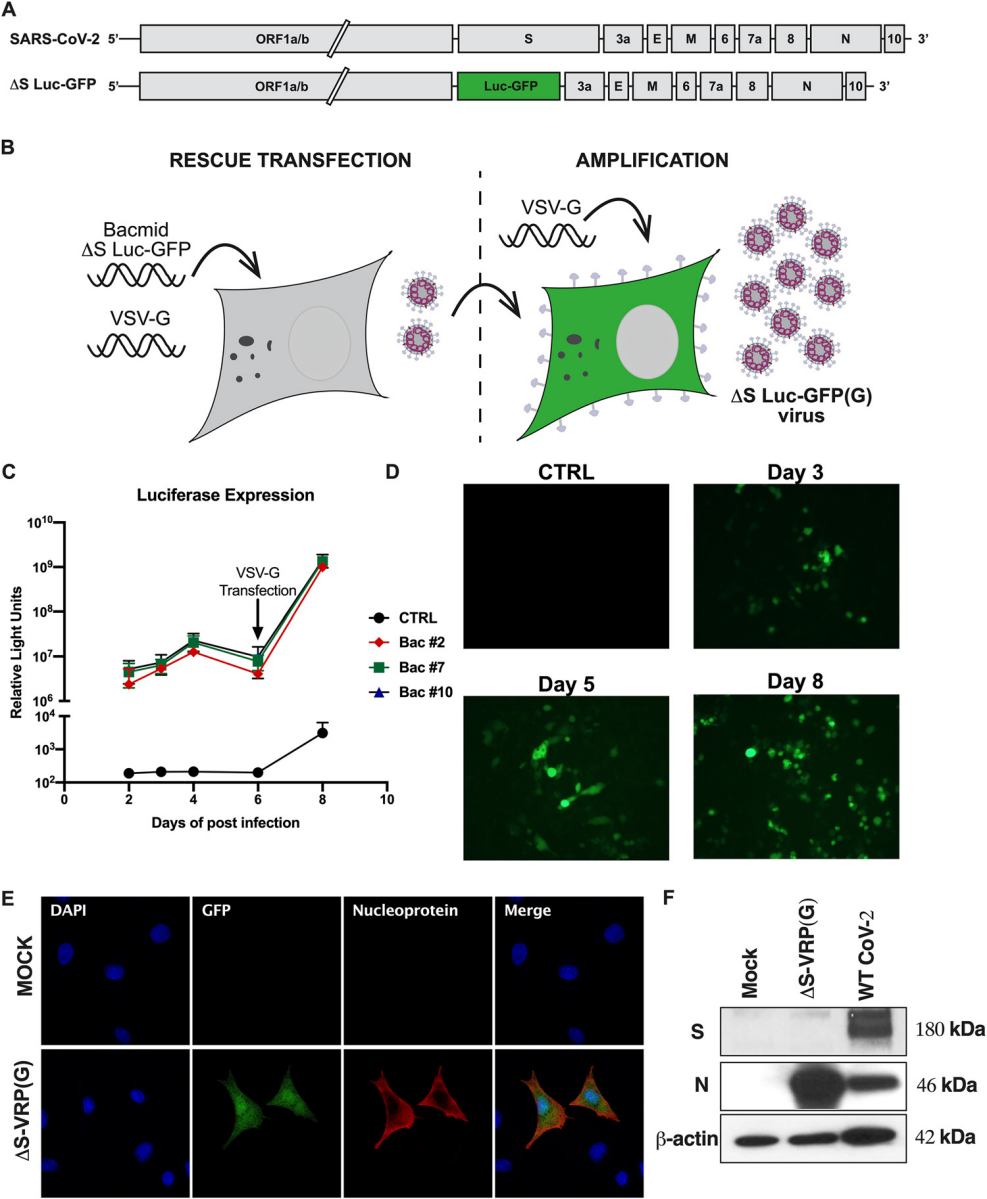
Development of single-cycle infectious SARS-CoV-2 replicon system with a dual reporter. (A) Schematic representation of SARS-CoV-2 and ΔS Luc-GFP SARS-CoV-2 genomes. The S gene in the SARS-CoV-2 genome was replaced with a luciferase and GFP dual reporter. (B) Generation and amplification of ΔS virus replicon particles. (Left) 293T/Huh7.5 cell mixture was transfected with ΔS Luc-GFP bacmid and VSV-G plasmid. (Right) Supernatants from ΔS-VRP(G) rescue transfections were amplified in Huh7.5 cells transfected with VSV-G plasmid. (C and D) Kinetics of luciferase and GFP expression during the rescue transfection process. 293T/Huh7.5 cells were cotransfected with ΔS Luc-GFP bacmid and VSV-G plasmid, and at various days posttransfection, luciferase activity in the supernatants and GFP expression in the cell mixture were assessed. On day 6 posttransfection, 293T/Huh7.5 cells were again transfected with additional VSV-G plasmid. Luciferase values (C) and GFP expression (D) are shown at the indicated time points. Luciferase activity is shown for 3 independent Bac clones. GFP expression is shown for Bac clone 7. (E) Immunofluorescence imagining of GFP and nucleoprotein expression in ΔS-VRP(G)-infected cells. A549-hACE2 cells were infected with ΔS-VRP(G), and at 18 hpi, cells were stained with anti-N antibody and imaged. (F) Western blot analysis of nucleoprotein expression in ΔS-VRP(G)-infected cells. A549-hACE2 cells were infected with ΔS-VRP(G), and at 18 hpi, cell lysates were collected and analyzed for nucleoprotein and spike protein expression by Western blotting. Cell lysates from wild-type SARS-CoV-2-infected cells were included as controls. β-Actin levels are shown as loading controls.

To safely study SARS-CoV-2 biology under BSL2 containment, we deleted and replaced the essential viral spike (S) ORF with a tandem *Gaussia* luciferase and neon GFP dual reporter separated by the porcine teschovirus 2A ribosome skipping signal under the control of the S gene transcription regulatory sequence (TRS) (ΔS-Luc-GFP [Fig. 1A]). Similar to other viral replicons, the ΔS-Luc-GFP genome can undergo normal transcription and replication upon transfection into cells, yet it is unable to produce infectious virus particles due to the lack of the S gene. Therefore, formation of infectious particles carrying the ΔS-Luc-GFP genome requires expression of the S protein or another viral glycoprotein(s) in producer cells. We rescued infectious VRPs by cotransfecting the ΔS-Luc-GFP bacmid with a VSV-G expression plasmid into a mixture of 293T/Huh7.5 cells [ΔS-VRP(G) [Fig. 1B]). We observed a significant increase in luciferase activity in the supernatants and GFP expression in cells transfected with ΔS-Luc-GFP bacmid over time compared to that in control vector-transfected cells (Fig. 1C and D). On day 6 posttransfection, the 293T/Huh7.5 cell mixture was transfected again with a VSV-G expression plasmid, which resulted in significant increases in both luciferase activity and numbers of GFP-expressing cells. These data show that ΔS-Luc-GFP replicated efficiently in cells, and additional expression of VSV-G presumably increased the spread of the ΔS-Luc-GFP genome to new cells. To produce ΔS-VRP(G) working stocks, supernatants from ΔS-VRP(G) rescue transfections were used as seed stocks to infect VSV-G-expressing Huh7.5 cells. Next, we evaluated the infectivity of amplified ΔS-VRP(G) stocks by measuring the expression of viral nucleoprotein (N) and GFP in a human lung epithelial cell line (A549) expressing human angiotensin-converting enzyme 2 (hACE2) (A549-hACE2). At 18 h postinfection (hpi), immunofluorescence analysis showed GFP expression in N-positive cells (Fig. 1E). Furthermore, Western blot analysis showed expression of N protein but not S protein in ΔS-VRP(G)-infected cells (Fig. 1F). Wild-type SARS-CoV-2-infected A549-hACE2 cell lysates were included as controls. Taken together, the results show that we have successfully established a SARS-CoV-2 VRP system with a dual reporter that can be easily propagated in VSV-G-expressing cells.

### ΔS-VRP(G) infection is restricted to a single cycle and neutralized by anti-VSV sera.

**FIG 2:**
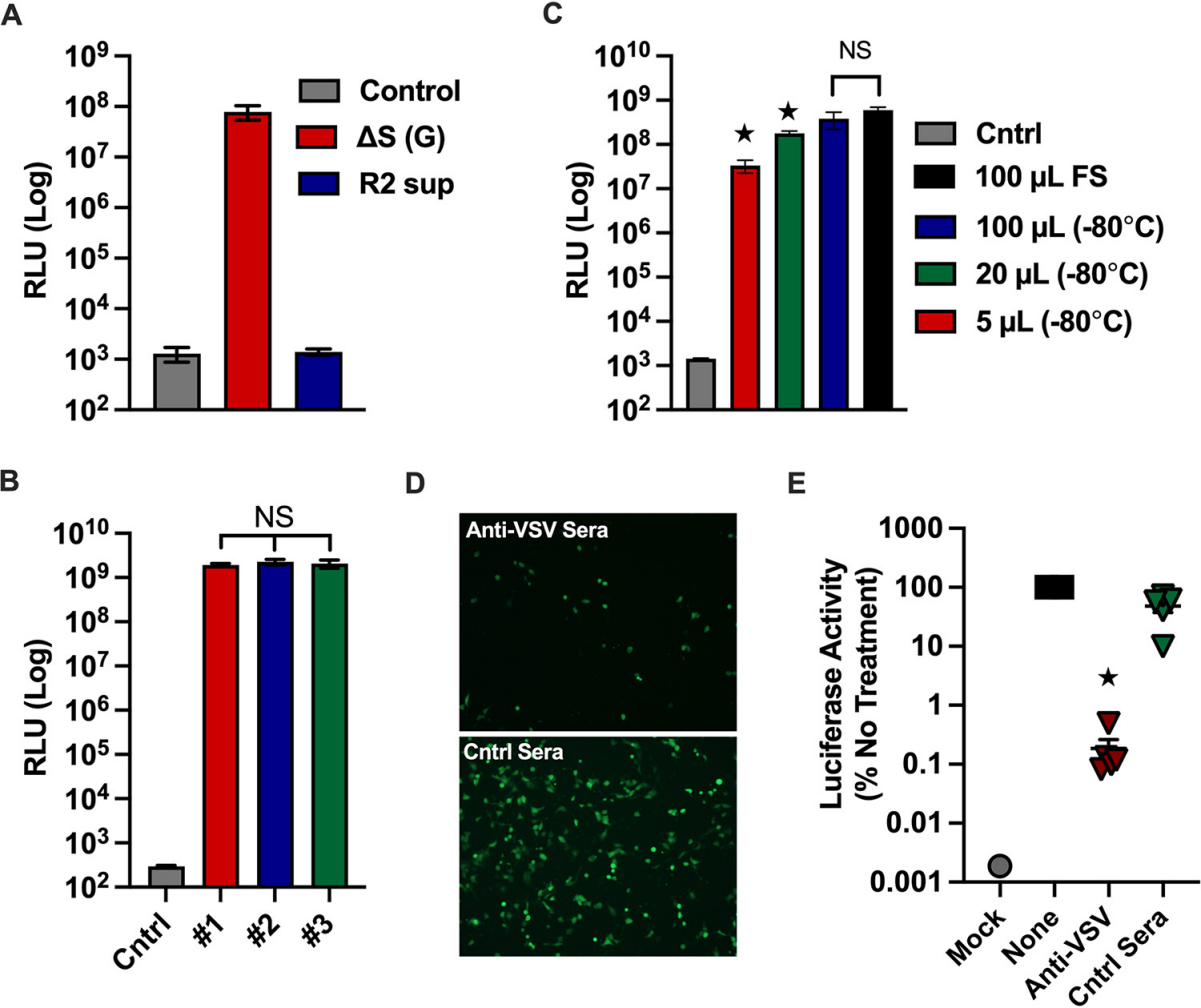
Characterization of ΔS-VRP dual reporter system. (A) ΔS-VRP(G) infection is restricted to a single round. Huh7.5 cells were infected with ΔS-VRP(G), and at 2 hpi, cells were washed and incubated in fresh medium. At 48 h, supernatants (round 2 [R2 sup]) were collected and added to new Huh7.5 cells. At 18 hpi, luciferase activity in the supernatant was measured. (B) Comparison of infectivity of different ΔS-VRP(G) preparations. Huh7.5 cells were infected with 3 independent preparations of ΔS-VRP(G), and luciferase activity was measured at 18 hpi. (C) Infectivity of ΔS-VRP(G) stored at −80°C. Huh7.5 cells were infected with ΔS-VRP(G) stored at −80°C or fresh preparations (FS), and luciferase activity was measured at 18 hpi. (D and E) Neutralization of ΔS-VRP(G) infection by anti-VSV sera. Sera from control (Cntrl) and VSV-infected mice were preincubated with ΔS-VRP(G) for 1 h and subsequently incubated with Huh7.5 cells for 2 h. Luciferase and GFP expression were assessed at 18 hpi. For panels A to C, luciferase activity in the supernatants was measured and is shown as relative light units (RLU). For panel E, luciferase activity was normalized to the no-treatment control and is shown as a percentage of the no-treatment control.

Next, we compared the infectivity of ΔS-VRPs produced in Huh7.5 cells expressing glycoproteins from diverse viral families, including SARS-CoV-2 S, and observed more efficient production of infectious ΔS-VRPs only in cells expressing VSV-G (data not shown); hence, our subsequent experiments were performed with ΔS-VRP(G) particles. Next, to demonstrate that infection from ΔS-VRP(G) is restricted to a single round, we transferred the supernatants from ΔS-VRP(G)-infected Huh7.5 cells (no VSV-G transfection [Fig. 2A, R2 sup]) onto fresh Huh7.5 cells and observed no GFP or Luc expression. These results demonstrate that infection with ΔS-VRP(G) is limited to a single cycle (Fig. 2A).

Next, to assess the reproducibility of the ΔS-VRP(G) system, we tested the infectivity of 3 independent preparations of ΔS-VRP(G) stocks in Huh7.5 cells and observed similar levels of luciferase activity across different preparations (Fig. 2B). In addition, ΔS-VRP(G) preparations were stable during storage at −80°C and retained infectivity at levels similar to those of fresh ΔS-VRP(G) preparations (Fig. 2C). Moreover, we observed a dose-dependent increase in the infectivity of ΔS-VRP(G) in Huh7.5 cells. Next, to demonstrate that ΔS-VRP(G) infection is mediated solely through the VSV-G glycoprotein, we tested if ΔS-VRP(G) infection can be inhibited in the presence of anti-VSV serum (Fig. 2D and E). Infectivity of ΔS-VRP(G) in Huh7.5 cells was significantly reduced in the presence of anti-VSV serum compared to control serum, indicating that ΔS-VRP(G) infection is mediated through the VSV-G protein. Taken together, the results show that we have developed a robust single-cycle infectious SARS-CoV-2 ΔS-VRP(G) dual reporter system that can be safely used under BSL2 containment.

### The ΔS-VRP(G) dual reporter system is a versatile tool to study SARS-CoV-2 biology in different cell types.

**FIG 3:**
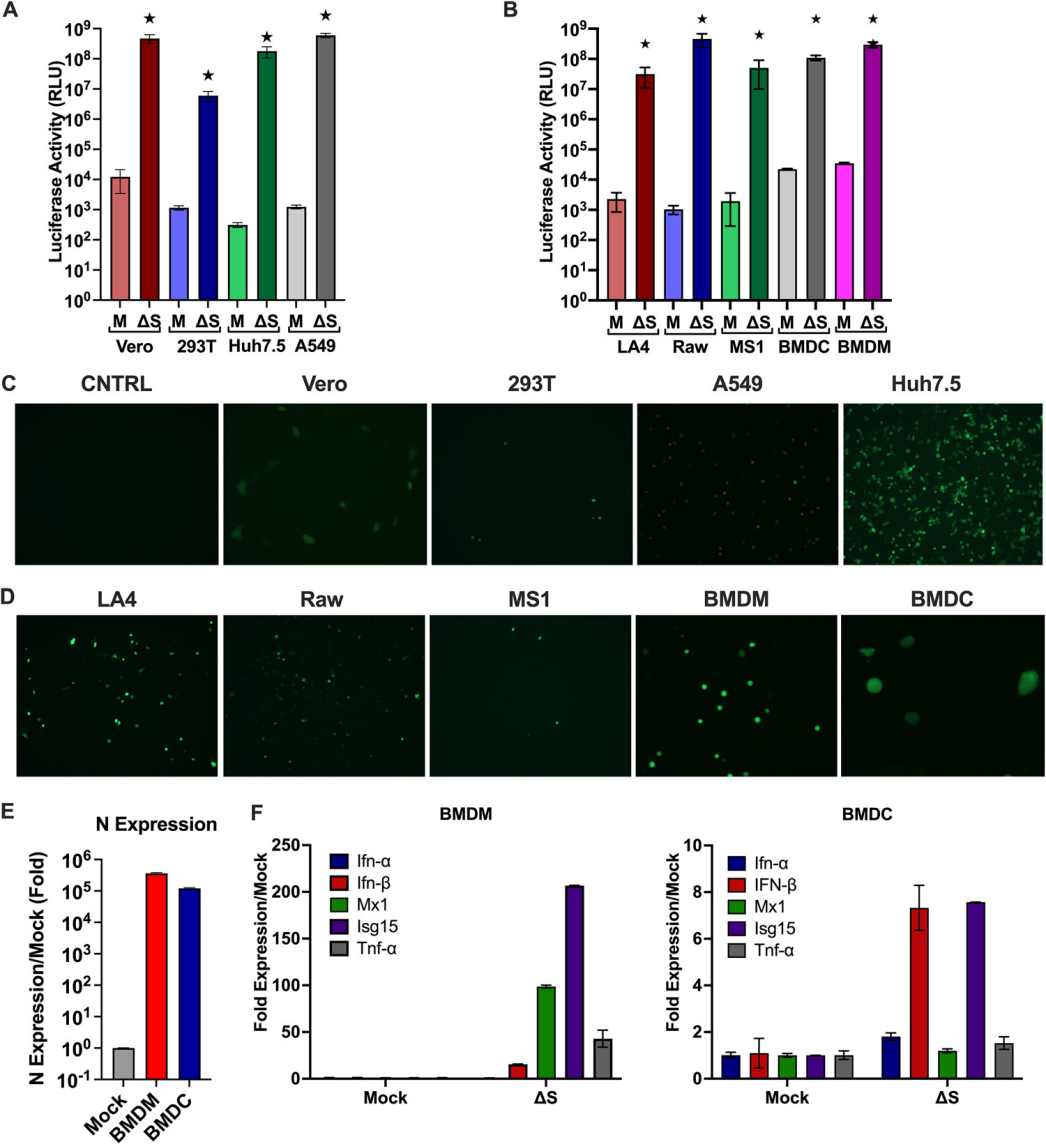
Permissiveness of human and murine cells to ΔS Luc-GFP replication. The indicated human and murine cells were infected with ΔS-VRP(G), and at 18 hpi, luciferase activity and GFP expression were measured. (A and B) Luciferase expression in human and murine cell lines. (C and D) GFP expression in human and murine cell lines. (E and F) Primary BMDM and BMDC were infected with ΔS-VRP(G), and at 18 hpi, expression levels of viral N mRNA and host antiviral genes were measured by qRT-PCR. (E) Viral N mRNA expression; (F) host antiviral gene expression.

As ΔS-VRP(G) infection of host cells is independent of SARS-CoV-2 host receptor ACE2 expression, we evaluated the robustness of the ΔS-VRP(G) dual reporter system in various cell types of human and murine origin (Fig. 3A to D). We observed robust replication of ΔS-Luc-GFP in various cell types from different species, including lung epithelial cells, kidney cells, monocytic cell lines, lymphocytes, macrophages, and dendritic cells. These results indicate that several human and murine cell types are permissive to replication of SARS-CoV-2. Interestingly, we observed differences in the levels of replication in different cells types, indicating that cell-specific host factors may regulate the intracellular replication steps of SARS-CoV-2.

Next, to investigate if the ΔS-VRP(G) dual reporter system can be useful for the assessment of host antiviral responses, we infected murine bone marrow-derived macrophages (BMDM) and dendritic cells (BMDC) and measured expression of the viral N gene and various host antiviral genes. We observed robust induction of various antiviral genes, including genes for alpha interferon (IFN-α), IFN-β, Mx1, Isg15, and tumor necrosis factor alpha (TNF-α) in BMDM and to a lesser extent in BMDC (Fig. 3E). Taken together, these results demonstrate that the ΔS-VRP(G) dual reporter system is a versatile tool to study SARS-CoV-2 biology as well as host immune responses in various cell types.

### The ΔS-VRP(G) dual reporter system can be valuable for antiviral drug screening.

**FIG 4:**
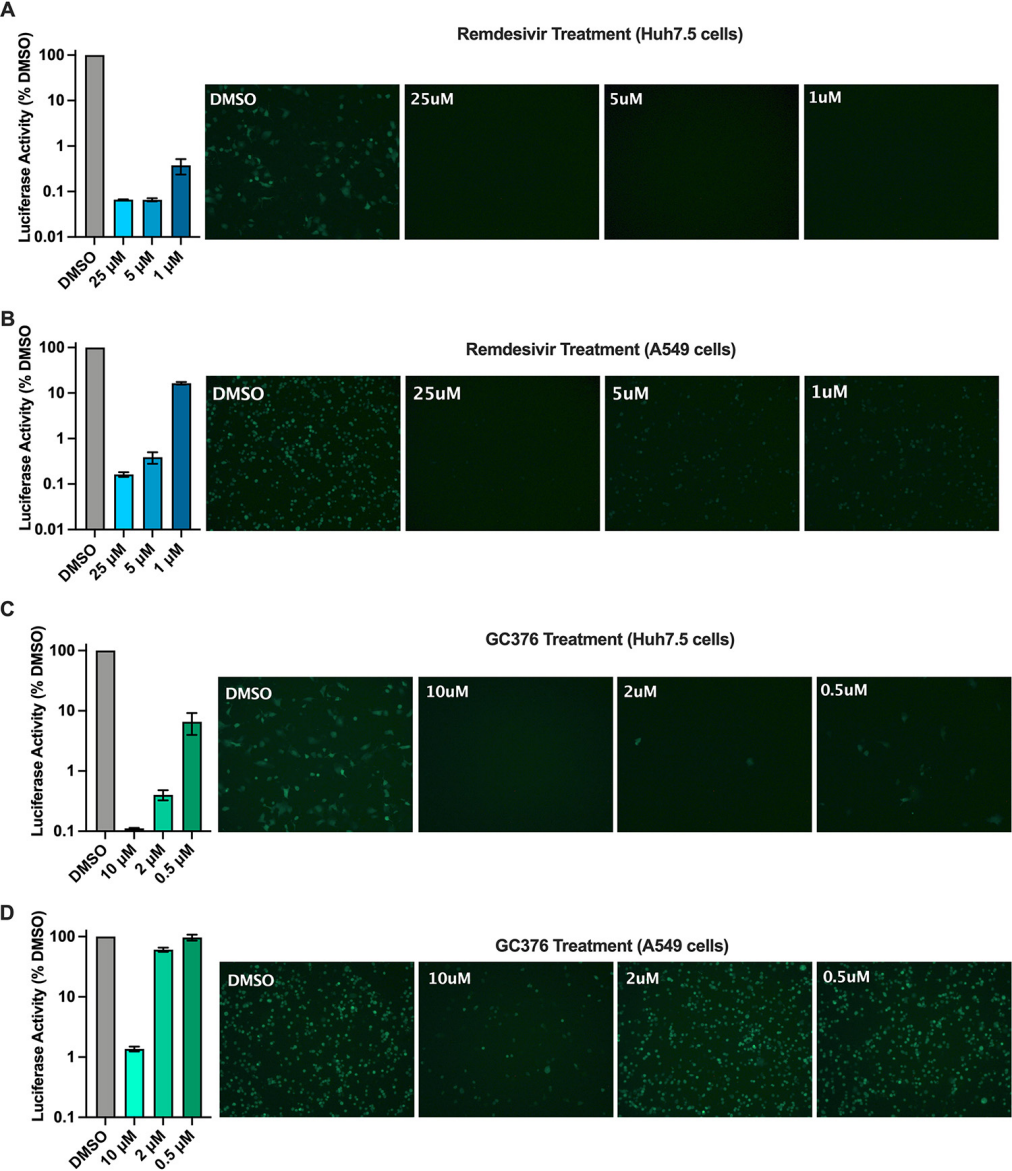
The ΔS-VRP(G) reporter system is suitable for antiviral drug screening. Huh7.5 or A549 cells were infected with ΔS-VRP(G) virus, and at 2 hpi, infected cells were treated with the indicated concentrations of remdesivir or GC376 dissolved in DMSO. At 18 hpi, GFP expression and luciferase activity were measured. (A and B) Assessment of effects of remdesivir treatment on ΔS Luc-GFP replication in Huh7.5 and A549 cells. (C and D) Assessment of effects of GC376 treatment on ΔS Luc-GFP replication in Huh7.5 and A549 cells. Luciferase values are normalized to DMSO control and shown as a percentage of DMSO control.

Next, to test if the ΔS-VRP(G) dual reporter system can be a useful platform for testing anti-SARS-CoV-2 drugs, we assessed the effects of the well-known anti-CoV drugs remdesivir and GC376 on ΔS-Luc-GFP replication (Fig. 4). Huh7.5 or A549 cells infected with ΔS-VRP(G) were treated with different concentrations of remdesivir or GC376 starting at 2 hpi. At 18 hpi, both luciferase activity in the supernatants and GFP expression in infected cells were compared between different treatment groups. In Huh7.5 cells, we observed a dose-dependent decrease in luciferase activity along with a concomitant decrease in GFP expression in both remdesivir and GC376 treatment groups compared to dimethyl sulfoxide (DMSO)-treated cells (Fig. 4A and C; remdesivir 50% inhibitory concentration [IC_50_], 26.7 nM, and GC376 IC_50_, 17.3 nM). We also observed a similar dose-dependent inhibition of ΔS-Luc-GFP replication in A549 cells treated with either remdesivir or GC376 (Fig. 4B and D; remdesivir, 55.2 nM, and GC376, 2,300 nM). Interestingly, inhibition of ΔS Luc-GFP replication by remdesivir and GC376 was less pronounced in A549 cells than in Huh7.5 cells, indicating cell-type-specific differences in the activity of antiviral drugs. These results demonstrate that the ΔS-VRP(G) dual reporter system can be useful for rapid screening of antiviral drugs against SARS-CoV-2 under BSL2 containment. Taken together, the results show that we have successfully established a SARS-CoV-2 VRP dual reporter platform that allows for safe investigation of SARS-CoV-2 biology, host interactions, and antiviral responses, as well as for high-throughput screening of anti-CoV drugs, under BSL2 containment.

## DISCUSSION

Studies with highly pathogenic viruses can be safely performed in biological laboratories with specialized biocontainment procedures. Through the generation of conditional replicating mutants or attenuated mutants, we can safely study some aspects of virus biology in standard BSL2 or BSL2+ facilities. Currently, research activities with SARS-CoV-2 are restricted to BSL3 facilities that mostly have limited capabilities. To overcome this limitation, we have developed a conditional mutant of SARS-CoV-2 through deletion and replacement of the essential viral S glycoprotein gene with a luciferase-GFP dual reporter, leaving the remaining SARS-CoV-2 genome intact. Through coexpression of VSV-G protein in *trans*, we have successfully generated single-cycle infectious SARS-CoV-2 replicon particles carrying the ΔS Luc-GFP genome. Various human and murine cells infected with ΔS-VRP(G) showed robust expression of both luciferase and GFP reporters, indicating that these cell lines are permissive to intracellular replication of SARS-CoV-2. In addition, as viral entry of ΔS-VRP(G) particles is mediated through the G protein, ΔS-VRP(G) can efficiently infect a variety of human and murine cell types lacking the SARS-CoV-2 ACE2 receptor. Importantly, the ΔS-VRP(G) dual reporter system showed robust responsiveness to treatment with remdesivir or GC376, demonstrating that the ΔS-VRP(G) dual reporter system can be useful for high-throughput screening of antiviral drugs against SARS-CoV-2.

Several groups have reported the development of SARS-CoV-2 replicon systems with either luciferase or GFP reporters, and the majority of these replicon systems require transfection of replicon DNA or RNA into cells, which may limit studies to cell types with higher transfection efficiency. In addition, there might be a time lag between nucleic acid transfection and optimal reporter expression. One advantage of our ΔS-VRP(G) dual reporter system is that large quantities of ΔS-VRP(G) can be easily amplified in VSV-G-expressing Huh7.5 cells and can be used to investigate SARS-CoV-2 biology in a variety of cell types. In addition to replicons, conditional SARS-CoV-2 mutants with GFP or luciferase reporters have been developed. Ju et al. generated a mutant lacking a portion of the essential N gene, thereby limiting replication to cells expressing N (14). Zhang et al. generated a conditional mutant lacking the ORF3a/E genes, which restricted replication to cells stably expressing ORF3a and -E proteins (13). As an additional safety measure, these authors elegantly modified all TRS sites to restrict the potential emergence of replication-competent virus through recombination with wild-type SARS-CoV-2. Another advantage of our ΔS-VRP(G) system is that VSV-G-mediated delivery of the ΔS-Luc-GFP genome occurs independently of the ACE2 receptor. Indeed, a vast majority of human and murine cell types supported ΔS-Luc-GFP replication, albeit with some differences in the levels of replication. It is possible that the observed variations in ΔS-Luc-GFP replication can be in part due to inherent differences in VSV-G-mediated entry into various cell types. Finally, as the ΔS-VRP(G) system contains both luciferase and GFP reporters, we observed a strong correlation between luciferase activity and GFP expression across different treatment groups in our studies with remdesivir and GC376. The presence of a dual reporter allows for rapid elimination of false-positive hits that directly inhibit luciferase or interfere with GFP fluorescence during high-throughput antiviral drug screening.

In our studies, we observed robust luciferase and GFP expression in cells infected with ΔS-VRP(G) compared to ΔS-VRP produced in cells expressing other viral glycoproteins, including SARS-CoV-2 S (data not shown). We speculate that this is in part due to the higher efficiency of VSV-G protein in mediating viral entry than for other viral glycoproteins. It is not completely clear how VSV-G is incorporated onto ΔS-VRPs to mediate infection. VSV-G has been reported to be localized to intracellular compartments, such as the endoplasmic reticulum-Golgi, as well as on the plasma membrane. As SARS-CoV-2 assembly and budding are thought to occur in the endoplasmic reticulum-Golgi intermediate compartment (ERGIC), it is possible that intracellularly expressed VSV-G is incorporated onto VRP membranes during canonical SARS-CoV-2 budding in the ERGIC. In agreement with VSV-G-dependent delivery of the ΔS Luc-GFP genome, ΔS-VRP(G) infection was neutralized in the presence of anti-VSV sera. Our future studies will determine if VSV-G incorporation onto ΔS-VRPs occurs in intracellular compartments.

In conclusion, we have developed a SARS-CoV-2 VRP platform with a dual reporter that can serve as a versatile tool to study SARS-CoV-2 host biology under BSL2 containment. Importantly, we observed robust and dose-dependent changes in both luciferase and GFP expression upon treatment with anti-CoV drugs, demonstrating that this ΔS-VRP(G) platform can be suitable for safe high-throughput screening of antivirals against SARS-CoV-2.

## MATERIALS AND METHODS

### Biosafety statement.

Studies with infectious SARS-CoV-2 and ΔS-VRP(G) viruses were approved by the UIowa IBC and CCOM BSL3 Oversight Committee (protocol number 210053). Initial validation and safety studies with ΔS-VRP(G) were performed in a BSL3 laboratory. After review of the safety data, both UIowa IBC and the NIH Office of Science Policy approved the use of ΔS-VRP(G) under BSL2+ containment at UIowa.

### Ethics statement.

All studies were performed in accordance with the principles described by the Animal Welfare Act and the National Institutes of Health guidelines for the care and use of laboratory animals in biomedical research. The protocol for isolating anti-VSV serum from mice was reviewed and approved by the Institutional Animal Care and Use Committee at the University of Iowa (animal protocol number 1062127).

### Anti-VSV serum.

Antiserum against VSV was produced in-house by immunizing C57BL/6J mice (6 weeks old) with 2 × 10^8^ PFU of live VSV-GFP via the intramuscular route. On day 14, mice were boosted with the same dose of VSV-GFP. At 4 weeks postimmunization, serum was collected by cardiac puncture and used in neutralization experiments after heat inactivation.

### Cell lines and primary cells.

Human lung epithelial cells (A549), human embryonic kidney cells (HEK293T), a hepatocellular carcinoma cell line (Huh7.5), a mouse endothelial cell line (MS1), mouse lung epithelial cells (LA4), a mouse macrophage line (Raw 264.7), and African green monkey kidney epithelial cells (Vero) were cultured in Dulbecco’s modified Eagle medium (DMEM) supplemented with 10% fetal bovine serum (FBS) and 1% penicillin/streptomycin (10,000 U/ml). Primary bone marrow-derived macrophages (BMDM) and dendritic cells (BMDC) were generated from bone marrows isolated from C57BL/6J mice (Jackson labs) by culturing in the presence of interleukin 4 (IL-4) and granulocyte-macrophage colony-stimulating factor (GM-CSF) or IL-4 and L929 conditioned medium, respectively (17, 18).

### Generation of ΔS-Luc-GFP bacmid.

A SARS-CoV-2 reverse genetics system based on the sequence of Wuhan-Hu-1/2019 isolate (GenBank accession number NC_045512) was designed and assembled into a pBeloBac11 vector as previously described for other coronaviruses (16). Our SARS-CoV-2 bacmid carries a unique engineered SanDI restriction site in the NSP15 gene and a naturally occurring unique BamHI restriction site near the 3′ end of the S gene (70 nucleotides before the ORF3a TRS). A gene fragment representing the dual reporter under the control of the S TRS was chemically synthesized as a 1.7-kb gBlock fragment (Integrated DNA Technologies) in the following in-frame arrangement of reporters: S gene TRS, S signal peptide (MFVFLVLLPLVSSQC), *Gaussia* luciferase, porcine teschovirus 2A site (ATNFSLLKQAGDVEENPG⇓P), and neon GFP reporter. The genomic sequence between the SanDI site and the S TRS was PCR amplified using PrimeStar Max (Clontech). The two fragments were combined by fusion PCR and subsequently cloned into the SARS-CoV-2 bacmid cut with SanDI and BamHI enzymes using the HiFi assembly system (New England BioLabs [NEB]). Assembly mixtures were transformed into DH10Bac, and individual ΔS-Luc-GFP bacmid clones were identified by restriction digestion of bacmid DNA.

### Rescue and amplification of ΔS-VRP(G).

To rescue recombinant ΔS-VRP(G) virus, a mixture of 293T cells/Huh7.5 cells in suspension (1 × 10^6^ cells of each cell type) were transfected with 4 μg of ΔS-Luc-GFP bacmid and 1 μg of VSV-G plasmid (Addgene; 138479) using polyethyleneimine (PEI) transfection reagent (Polysciences; DNA/PEI ratio, 1:4). After 5 h posttransfection, the transfection mixture-containing medium was replaced with DMEM/2% FBS. At 72 to 96 h posttransfection, supernatants were collected and kept as ΔS-VRP(G) seed stocks. To amplify ΔS-VRP(G), Huh7.5 cells seeded in 150-mm plates (1.5 × 10^7^) were transfected with 40 μg of VSV-G plasmid using PEI reagent. At 5 h posttransfection, the transfection mixture-containing medium was replaced with DMEM/10% FBS. On the following day, 1 mL of ΔS-VRP(G) seed stock was added to VSV-G transfected Huh7.5 cells and incubated for 2 h. After a washing with phosphate-buffered saline (PBS), infected Huh7.5 cells were placed in 25 mL of DMEM/2% FBS and monitored for GFP expression and cytopathic effects (CPE). At 48 to 72 h postinfection, supernatants were collected, clarified of debris, aliquoted, and stored at −80°C. These stocks were used to perform subsequent infection experiments.

### ΔS-VRP(G) infection.

Indicated cell types were seeded in 12-well (1  × 10^5^ to 2 × 10^5^ per well) or 6-well (5 × 10^5^ to 8 × 10^5^ per well) plates a day prior to infection and infected with 0.5 mL or 1 mL of ΔS-VRP(G) stock, respectively. After 2 h of incubation, the inoculum was removed and replaced with DMEM/2% FBS after two PBS washes. At 18 hpi, luciferase activity in the supernatant and GFP expression in the cells were measured. For measurement of luciferase activity, 50 μL of supernatant was mixed with 50 μL of *Renilla* luciferase substrate (Promega) and immediately measured in a GloMax 20/20 single-tube luminometer. GFP images were captured with a Nikon Eclipse microscope using a 20× objective at an exposure time of 600 ms.

### Remdesivir and GC376 inhibition.

A549 or Huh7.5 cells seeded at a density of 1.5 × 10^5^ per well (12-well plate) a day prior were infected with 0.5 mL of ΔS-VRP(G) stock. After 2 hpi, cells were washed with PBS and placed in DMEM/2% FBS with the indicated amounts of drug. Measurement of luciferase activity and GFP expression were performed as described above at 18 hpi.

### Immunofluorescence.

A day prior to infection, A549 cells were seeded onto glass coverslips at 1 × 10^5^/well in a 24-well plate in Opti-modified Eagle medium (Opti-MEM) and infected with 0.5 mL of ΔS-VRP(G). After 2 h, viral inoculum was replaced with Opti-MEM. At 18 hpi, infected cells were washed twice with PBS and fixed with 4% paraformaldehyde (Electron Microscopy Sciences) in PBS for 5 min at room temperature. After a washing with PBS, fixed cells were permeabilized with 0.3% Triton X-100 in PBS for 5 min, washed with PBS, and incubated in a blocking buffer consisting of 1% bovine serum albumin (BSA), 0.5% fish gelatin (Sigma), and 0.01% Tween 20 (Sigma) in PBS for 30 min. Permeabilized cells were incubated in blocking buffer with mouse anti-nucleoprotein antibody (1:1,000; Sino Biological) for 1 h, followed by staining with goat anti-mouse Alexa Fluor 647 secondary antibody for 30 min. Coverslips were mounted onto microscopy slides with ProLong Gold antifade mountant with 4′,6-diamidino-2-phenylindole (DAPI) (Invitrogen). All incubations involving antibody staining were performed with gentle shaking at room temperature. Images were acquired on a Leica DFC7000T microscope under a 63× oil immersion objective using Leica software and processed using ImageJ software.

### Western blot analysis.

A549 cells seeded in 6-well plates at 1 × 10^6^/well were infected with 1 mL of ΔS-VRP(G), and at 18 hpi, cells were lysed in radioimmunoprecipitation assay (RIPA) buffer and protein samples were separated on a 4 to 15% gradient SDS-PAGE gel (Bio-Rad). As controls, A549-hACE2 cells were infected with SARS-COV-2 (Wuhan-Hu-1/2019) at a multiplicity of infection (MOI) of 3 and lysed in RIPA buffer. Western blot analysis was performed following the transfer of proteins onto a nitrocellulose membrane using mouse anti-nucleoprotein or S antibody (1:1,000; Sino Biological) and goat anti-mouse secondary antibody conjugated to horseradish peroxidase (GENA931; Sigma).

### Quantitative RT-PCR analysis.

BMDM and BMDC seeded at a density of 2 × 10^6^ cells/well were infected with 2 mL of ΔS-VRP(G), and at 18 hpi, total RNAs from infected cells were extracted using a PureLink RNA extraction kit according to the manufacturer’s instructions. Residual genomic DNA contamination was removed by DNase I (Invitrogen; catalog number 12185010) treatment. cDNA was synthesized using Superscript IV reverse transcriptase (RT; Invitrogen; catalog number 18090010) and oligo(dT) (Invitrogen), and quantitative PCR (qPCR) analysis was performed using SYBR green PCR master mix (Applied Biosystems; catalog number 4368702) with technical duplicates and gene-specific primers (19). 18S RNA was used as an endogenous housekeeping gene to calculate delta cycle thresholds. qPCR primers were as follows: SARS-CoV-2 N, forward, CAATGCTGCAATCGTGCTAC, and reverse, GTTGCGACTACGTGATGAGG; mouse IFN-α1, forward, TCAAAGGACTCATCTGCTGCTTG, and reverse, CCACCTGCTGCATCAGACAAC; mouse IFN-β, forward, CAGCTCCAAGAAAGGACGAAC, and reverse, GGCAGTGTAACTCTTCTGCAT; Mx1, forward, GACCATAGGGGTCTTGACCAA, and reverse, AGACTTGCTCTTTCTGAAAAGCC; Isg15, forward, GGTGTCCGTGACTAACTCCAT, and reverse, TGGAAAGGGTAAGACCGTCCT; TNF-α, forward, GACGTGGAACTGGCAGAAGAG, and reverse, TTGGTGGTTTGTGAGTGTGAG; and 18S RNA, forward, AAACGGCTACCACATCCAAG, and reverse, CCTCCAATGGATCCTCGTTA. Results are represented as fold expression relative to that in mock samples.

### Statistical analysis.

Significance of data points was assessed using the unpaired Student *t* test. In figures, a star indicates a *P* value of <0.05 and “NS” indicates nonsignificance.
